# Increasing obstructive sleep apnea risk is associated with hearing impairment in middle-aged Chinese men—A cross-sectional study

**DOI:** 10.1371/journal.pone.0268412

**Published:** 2022-05-20

**Authors:** Yu Li, Xiaoyi Wang, Jing Cui, Jiping Ren, Zhong Xin, Dongning Chen

**Affiliations:** 1 Physical Examination Department, Beijing Tongren Hospital, Capital Medical University, Beijing, China; 2 Department of Otolaryngology, Beijing Tongren Hospital, Capital Medical University, Beijing, China; 3 Department of Endocrinology, Beijing Tongren Hospital, Capital Medical University, Beijing, China; Sapienza University of Rome, ITALY

## Abstract

**Objective:**

Midlife males with obstructive sleep apnea (OSA) bear a high risk for cardiovascular diseases. However, the association of OSA and hearing impairment is controversial. Our objective was to observe the incidence of hearing loss in middle-aged males with different risks for OSA.

**Methods:**

794 men aged 40–65 who participated in health examination and pure tone hearing screening between January and June 2021 were recruited in the study. Medical history was collected. Height, weight and blood pressure were tested, and biochemical test including blood lipids and blood glucose was performed. According to the STOP-BANG score, the observed subjects were divided into low, intermediate and high groups for OSA risk. Hearing impairment was defined as failure in responding to any pure tone of 25 dB HL in any ear at the frequencies: 4 kHz for high frequency range and 0.5k, 1k, 2 kHz for low/medium frequency range. The incidence of hearing loss in those groups was compared after adjusting the cardiovascular risk factors.

**Results:**

The incidence of hearing impairment in the groups of intermediate, high, and intermediate/high risk for OSA (46.9%, 45.2%, 46.3%, respectively) were higher than that in the group of low risk for OSA (33.3%, P<0.001). After adjusting cardiovascular risk factors, the risk of hearing impairment in the group of high risk for OSA is 1.64 times of the group of low risk for OSA (95%CI: 1.02–2.69, P<0.05). The risk of hearing impairment at high frequency(4kHz) in the group of intermediate/high risk for OSA is 1.43 times of the group of low-risk for OSA (95%CI: 1.00–2.06, P<0.05).

**Conclusion:**

The risk of hearing impairment in midlife men with high, intermediate/high risk for OSA is significantly increased, especially at high frequency of 4 kHz.

## Introduction

Obstructive sleep apnea (OSA) is characterized by recurrent upper airway collapse during sleep, leading to sleep apnea and intermittent hypoxia. It is considered to be a risk factor for a variety of chronic diseases including hypertension, diabetes, ischemic coronary heart disease, stroke, cancer and cognitive impairment [1]. A study showed that moderate/severe OSA is a risk factor for incident major adverse cardiovascular and cerebrovascular events (MACCE) [2]. Neurocognitive function and the risk of developing neurodegenerative diseases are also associated with the presence and severity of OSA. The prevalence of OSA in Chinese males is 5.7%, and increases with age, with a peak at age of 40–69 [3]. Chinese males are more susceptible to obesity, resulting in a high OSA prevalence and related health problems that deserve more attention [1]. Pathophysiologic influences of OSA involve increased systemic inflammation, autonomic nerve system alterations, oxidative stress, enhanced prothrombotic state, and vascular dysfunction [4]. Nowadays, some smartphone applications (Apps) in Otolaryngology have been used for early diagnosis of OSA [5]. Among the treatments currently available for OSA, surgery in selected patients can represent excellent results both on the improvement of neurocognitive performance and apnea indexes [6].

As the fourth leading cause for disability, hearing impairment has been focused since it may lead to disturbed interpersonal communication, social loneliness and cognitive impairment. A Study showed that 23% of people aged 12 years or older were suffering from hearing loss [7]. The known risk factors for hearing loss include age, noise, ototoxic drugs, smoking, diabetes, dyslipidemia, coronary heart disease, and so on [8].

Recently, it has been reported that OSA was related to hearing impairment. However, the results are inconsistent. Vorlova et al [9] showed a decreased perception of high frequency in sever OSA. In contrast, Hwang et al [10] did not find positive association between OSA and averaged pure-tone threshold neither of low nor of high frequencies. The purpose of this study was to observe the association of risks for OSA assessed by STOP-BANG questionnaire with hearing impairment in middle-aged Chinese men.

## Materials and methods

### Study design and research population

**Fig 1:**
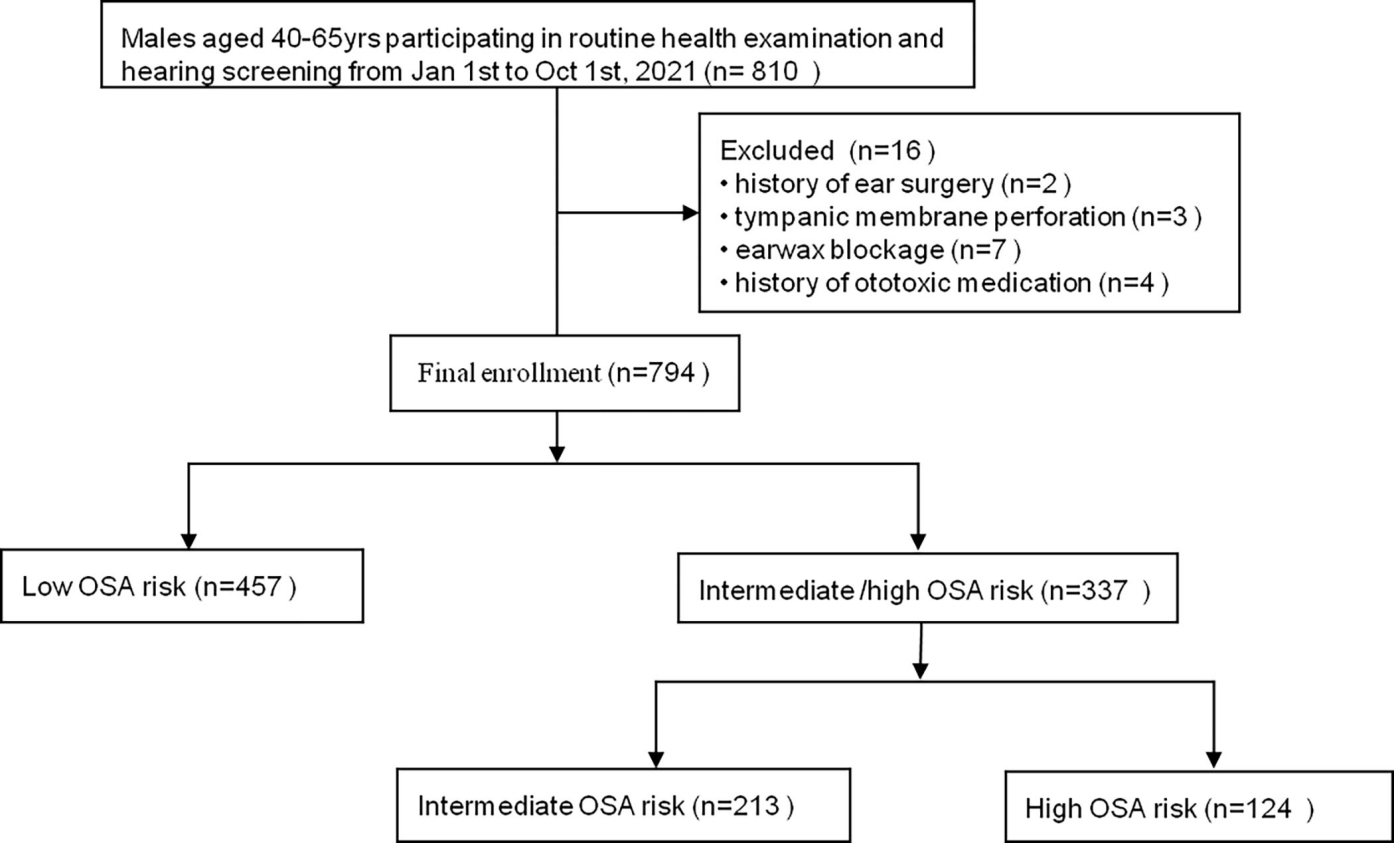
Description of the inclusion and exclusion procedure for enrollment of the research cohort.

Between January and October 2021, the cross-sectional, population based research was conducted in Beijing Tongren Hospital. Among the individuals who participated in routine health examination and received hearing screening, 794 men aged from 40 to 65 were recruited (Fig 1). Questionnaires related to medication history and STOP-BANG questions were completed beforehand. Blood samples were taken after eight hours’ fasting, followed by the measurement of height, weight and blood pressure. Pure tone hearing screening was performed.

The study was approved by the Ethics Committee of Beijing Tongren Hospital, Capital Medical University. All procedures were performed in the study in accordance with the 1964 Helsinki declaration and its later amendments. Informed written consents were obtained from all participants included in this study.

### Measurement of body mass index (BMI), neck circumference (NC) and biochemical indicators

BMI was derived by weight (kg) / height^2^ (m^2^). NC was measured at the horizontal level of the seventh cervical vertebra. Blood pressure was measured twice at the sitting position after resting for 5 min, and the average was used for analysis. Fasting blood samples were detected within 24 hours. Fasting blood glucose (FBG), creatinine, triglyceride (TG), total cholesterol (TC) and low density lipoprotein cholesterol (LDL) were measured by Immunoassay Systems (Beckman AU5821, USA). CCR (creatinine clearance rate, ml/min) = [(140-age) ×body weight (kg)] /serum creatinine (μmol/L) [11].

### OSA risk assessment

OSA risk was assessed by the STOP-BANG questionnaire [12] which contains eight questions with yes/no answers, including: 1. frequent snoring (S), 2. daily fatigue (T), 3. observed apnea (O), 4. hypertension (P), 5. BMI > 35 kg / m^2^ (B), 6. age > 50 years old (A), 7. neck circumference > 40 cm (N), and 8. male (G). Low risk = yes to 0–2 questions; high risk = yes to ≥ 5 STOP-BANG questions, or yes to ≥ 2 STOP questions plus neck circumference > 40 cm; intermediate risk = yes to 3–4 STOP-BANG questions with exclusion of those included in the high risk group.

### Hearing screening

Pure tone hearing screening was conducted in an independent quiet room with ambient noise less than 40 dB, using a AA20 screening audiometer (MACIO, Germany) and a Sennheiser HDA200 noise-masked headphone (Hannover, Germany). Testing was completed at frequencies of 0.5 kHz, 1 kHz, 2 kHz and 4 kHz for each ear separately. Otolaryngology and otoscopy examination were performed by experienced otolaryngologists before hearing screening test. Definition of hearing screening results: a pass was defined as response in both ears to pure-tone stimuli of 25 dB HL at all frequencies. A fail was defined as no response to 25 dB HL at any frequency in either ear. Hearing impairment in the Low/medium frequency range was defined as a fail at 0.5k, 1k or 2 kHz. Hearing impairment in the high frequency range was defined as a fail at 4 kHz [13].

### Statistical analysis

The Pearson χ^2^ test was used for categorical data, one-way ANOVA or independent t-test was used to compare continuous data. The missing values were filled with the median of the non-missing values. Sensitivity analyses were further carried out by assessing whether the present finding was influenced by compositions of subjects. Subjects in the intermediate/high, intermediate and high risk groups were analyzed respectively. The incidence of hearing impairment was shown as OR and 95%CI by binary logistic regression analysis. Different adjustment models were used to evaluate the incidence of hearing impairment in each OSA risk group. Model 1: no factors were adjusted. Model 2: age and BMI were adjusted. Model 3: model 2 plus smoking status were adjusted. Model 4: model 2 plus cardiovascular risk factors (hypertension, diabetes and dyslipidemia) were adjusted. For the multivariate logistic regression, the continuous data were transformed into categorical data: (1) age: ≤50 and > 50; (2) BMI: < 28 and ≥28kg/m^2^; (3) hypertension: no self-reported hypertension with SBP < 140 and DBP < 90mmHg vs otherwise; (4) diabetes: no self-reported diabetes with FBG < 7mmol/L vs otherwise; (5) dyslipidemia: no self-reported dyslipidemia with TG < 1.7mmol/L, TC < 5.2mmol/L and LDL < 3.4mmol/L vs otherwise. P values < 0.05 were considered statistically significant. SPSS 13.0 software was used for statistical analysis.

## Results

### Characteristics of the study population

A total of 794 middle—aged males enrolled in the study were grouped according to the STOP-BANG score. 457 (57.6%) had scores representing low risk for OSA. 213 (26.8%) had scores of intermediate risk, and 124 (15.6%) had scores of high risk for OSA. Together, the intermediate/high risk group added up to 337 (42.4%) of the study subjects. Subjects in the intermediate, high and intermediate/high risk groups tended to be older, and had higher BMI and higher proportion of hypertension, dyslipidemia and smokers. They also had a higher level of SBP, DBP, FBG, TG and CCR (Table 1). The components of STOP-BANG scores in each risk group were shown in Table 2. The missing data for each variable was no more than 20%.

**Table 1:** Characteristics of study population grouped by OSA risk assessed by STOP-BANG scores.

	Total (N = 794)	Low (N = 457)	Intermediate (N = 213)	High (N = 124)	P value^a^	Intermediate/high (N = 337)	P value^b^
Age	48.0±6.5	46.2±5.8	50.9±6.8	49.2±6.5	<0.001^¥^	50.3±6.7	<0.001*
BMI(kg/m^2^)	25.5±3.2	24.3±2.5	26.5±3.1	28.3±3.1	<0.001^¥^	27.2±3.2	<0.001*
Hypertension	175(26.3%)	16(3.5%)	73(34.3%)	86(69.4%)	<0.001^#^	159(47.2%)	<0.001^#^
Diabetes	32(4.0%)	16(3.5%)	9(4.2%)	7(5.6%)	0.552	16(4.7%)	0.377
Dyslipidemia	173(21.8%)	67(14.7%)	56(26.3%)	50(40.3%)	<0.001^#^	106(31.5%)	<0.001^#^
Ever smoker	324(40.8)	156(34.1%)	97(45.5%)	71(57.3%)	<0.001^#^	168(49.9%)	<0.001^#^
Smoker	9(1.1%)	2(0.4%)	5(2.3%)	2(1.6%)		7(2.1%)	
SBP (mmHg)	134±15	130±15	138±15	141±14	<0.001^¥^	139±15	<0.001*
DBP (mmHg)	86±11	83±11	88±10	92±10	<0.001^¥^	90±10	<0.001*
FPG (mmol/L)	6.0±1.5	5.8±1.2	6.2±1.8	6.5±2.1	<0.001^¥^	6.3±1.9	<0.001*
TG(mmol/L)	1.9±1.7	1.7±1.5	1.9±1.6	2.7±2.5	<0.001^¥^	2.2±2.0	<0.001*
TC (mmol/L)	5.1±1.0	5.2±0.9	5.1±1.1	5.0±1.1	0.358	5.1±1.1	0.389
LDL (mmol/L)	3.2±0.9	3.2±0.8	3.2±0.9	3.0±0.9	0.036^¥^	3.1±0.9	0.155
CCR (mL/min)	105.6±22.4	101.6±19.5	108.1±22.4	116.4±27.9	<0.001^¥^	111.2±24.9	<0.001*

P value^a^: comparing low vs intermediate and high.

P value^b^: comparing low vs intermediate/high.

^¥^: P<0.05, by one way ANOVA test

*: P<0.05, by independent T test

^#^: P<0.05, by Pearson χ^2^ test.

BMI: body mass index; SBP: systolic blood pressure; DBP: diastolic blood pressure; FPG: fasting blood glucose; TG: triglycerides; TC: total cholesterol; LDL: low-density lipoprotein cholesterol; CCR: creatinine clearance rate.

**Table 2:** Components of the STOP-BANG questionnaire grouped by OSA risk assessed by STOP-BANG scores.

	Total (N = 794)	Low (N = 457)	Intermediate (N = 213)	High (N = 124)	P value^a^	Intermediate/high (N = 337)	P value^b^
STOP-BANG scores							
Mean±SD	2.9±2.1	1.5±0.5	3.2±0.5	7.0±1.8	<0.001^¥^	4.6±2.2	<0.001*
STOP-BANG components	n(%)	n(%)	n(%)	n(%)		n(%)	
Snore loudly (S)	255(32.1%)	54(11.8%)	95(44.6%)	106(85.5%)	<0.001^#^	201(59.6%)	<0.001^#^
Daytime tiredness (T)	185(23.3%)	48(10.5%)	67(31.5%)	70(56.5%)	<0.001^#^	137(40.7%)	<0.001^#^
Observed apnea (O)	69(8.7%)	2(0.4%)	16(7.5%)	51(41.1%)	<0.001^#^	67(19.9%)	<0.001^#^
Hypertension (P)	175(26.3%)	16(3.5%)	73(34.3%)	86(69.4%)	<0.001^#^	159(47.2%)	<0.001^#^
BMI>35kg/m^2^ (B)	5(0.6%)	0	2(0.9%)	3(2.4%)	0.008^#^	5(1.5%)	0.014^#^
Age>50 (A)	292(36.8%)	111(24.3%)	126(59.2%)	55(44.4%)	<0.001^#^	181(53.7%)	<0.001^#^
NC>40cm (N)	355(44.7%)	113(24.7%)	125(58.7%)	117(94.4%)	<0.001^#^	242(71.8%)	<0.001^#^
Male (G)	794(100%)	457(100%)	213100%)	124(100%)		794(100%)	

P value^a^: comparing low vs intermediate and high.

P value^b^: comparing low vs intermediate/high.

^¥^: P<0.05, by one way ANOVA test

*: P<0.05, by independent T test

^#^: P<0.05, by Pearson χ^2^ test.

BMI: body mass index, NC: neck circumstance.

#### Comparison of hearing screening results among different groups of OSA risks

**Fig 2:**
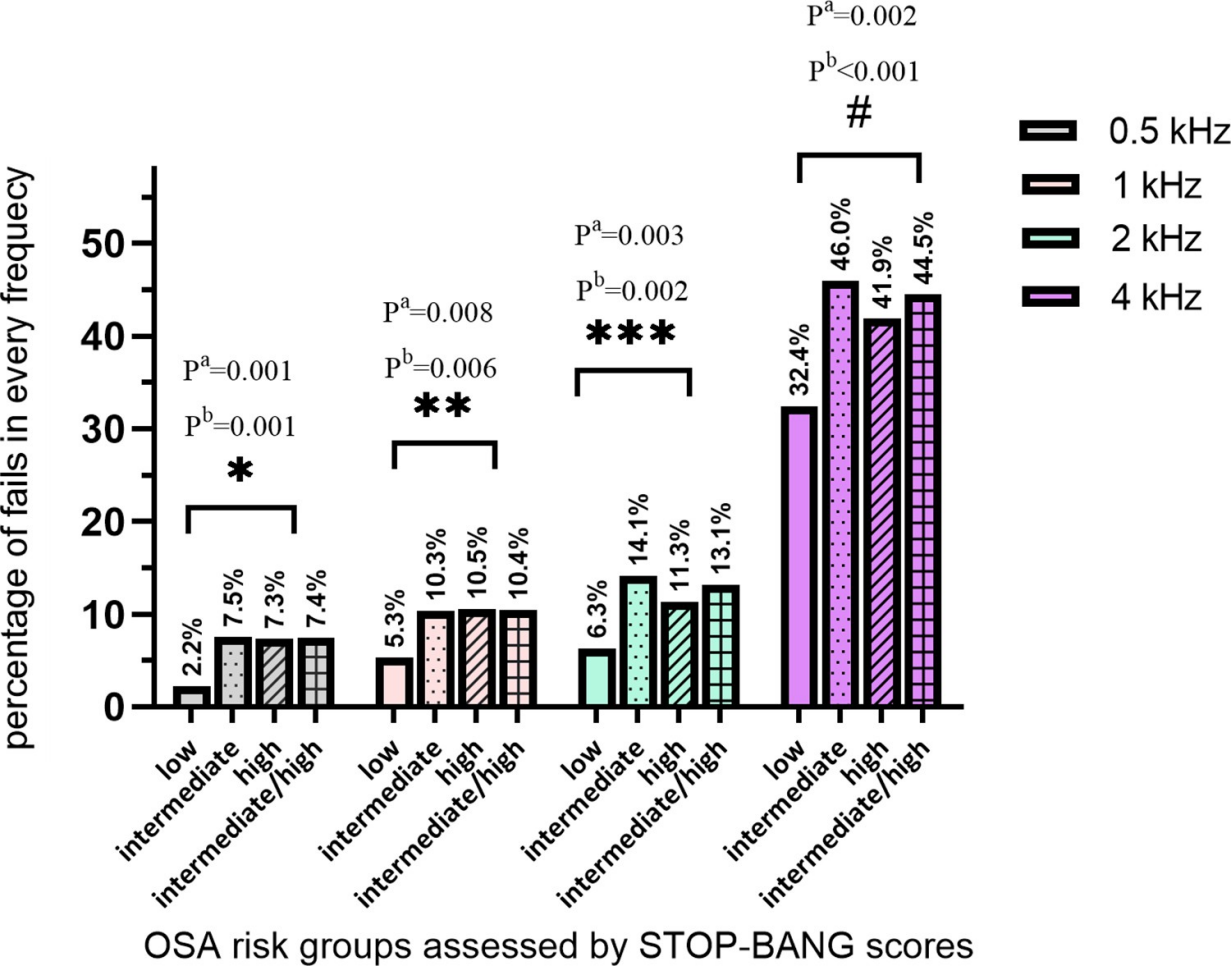
Percentage of pure-tone fails in all screening frequencies grouped by OSA risk based on STOP-BANG scores. P value^a^: comparing low vs intermediate and high. P value^b^: comparing low vs intermediate/high. *, **, ***, ^#^: P<0.05, by Pearson χ^2^ test. Plain columns: low OSA risk, columns with dots: intermediate OSA risk, columns with stripes: high OSA risk, columns with squares: intermediate/ high OSA risk.

The percentage of individuals who failed the pure-tone screening in the groups of intermediate, high and intermediate/high risk for OSA were higher than that in the group of low risk (Table 3). No significant difference was shown when comparing the otological risk factors between the intermediate/high risk group and the low risk group, nor in intermediate, high and low risk groups of OSA. Individuals with intermediate, high and intermediate/high risk for OSA had a higher percentage of hearing screening failure at 0.5, 1, 2, and 4 kHz. The hearing impairment at 4 kHz was the most significant than other frequencies (Fig 2).

**Table 3:** Comparing pure-tone screening fails and otologic risk factors grouped by OSA risk based on STOP-BANG scores.

	Total (N = 794)	Low (N = 457)	Intermediate (N = 213)	High (N = 124)	P value^a^	Intermediate/high (N = 337)	P value^b^
Pure-tone screening fails	n(%)	n(%)	n(%)	n(%)		n(%)	
At any ear	308(38.8%)	152(33.3%)	100(46.9%)	56(45.2%)	0.001^#^	156(46.3%)	<0.001^#^
At unilateral ear	112(14.1%)	57(12.5%)	36(16.9%)	19(15.3%)	0.007^#^	55(16.3%)	0.001^#^
At bilateral ears	196(24.7%)	95(20.8%)	64(30.0%)	37(29.8%)		101(50%)	
Otological questionnaire							
tinnitus	52(6.5%)	25(5.5%)	16(7.5%)	11(8.9%)	0.331	27(8.0%)	0.16
dizziness	3(0.9%)	2(0.4%)	0	1(0.8%)	0.483	1(0.3%)	0.744
Work noise exposure	15(1.9%)	6 (1.3%)	6(2.8%)	3(2.4%)	0.378	9(2.7%)	0.17

P value^a^: comparing low vs intermediate and high.

P value^b^: comparing low vs intermediate/high.

^#^: P<0.05, by Pearson χ^2^ test.

### Correlation between hearing impairment and the risk for OSA

**Fig 3:**
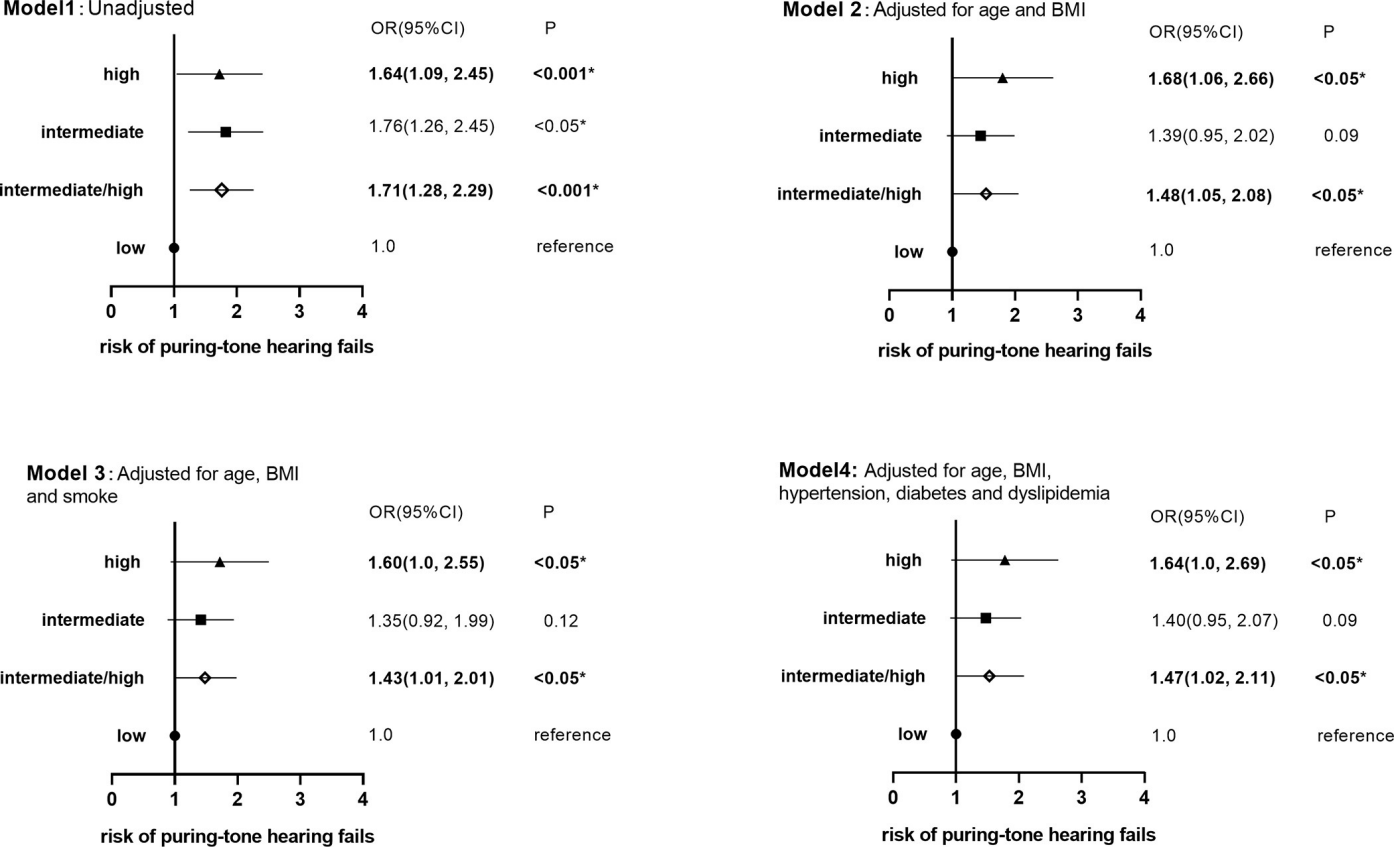
Multivariable models comparing the risk of pure-tone hearing fails grouped by OSA risk based on STOP-BANG scores. *: P≤0.05 (Model 2 adjusted for age and BMI. Model 3 adjusted for age, BMI and smoke. Model 4 adjusted for age, BMI, hypertension, diabetes and dyslipidemia.) Bold characters indicated statistical significance.

The incidence of hearing impairment at any frequency of any ear: The incidence of hearing impairment in the groups of intermediate, high and intermediate/high risk for OSA was significantly higher as compared to that of low risk (Fig 3, Model 1). After adjusting age and BMI (Fig 3, Model 2), age, BMI plus cardiovascular risk factors (smoking, hypertension, diabetes and dyslipidemia; Fig 3, Model 3 and 4), the incidence of hearing impairment in individuals with high and intermediate/high risk was significantly higher than that with low risk for OSA.

### The incidence of hearing impairment at different frequencies

After adjusting age and BMI, and age, BMI plus cardiovascular risk factors, the incidence of hearing impairment at high frequency in individuals with intermediate/ high risk for OSA was significantly higher than that with low risk (Table 4). Whereas no significant difference was shown as for the hearing impairment in the low/medium frequency range, between the group of intermediate/ high and low risk for OSA after adjusting confounding factors.

**Table 4:** Multivariable models comparing the risk of pure-tone hearing fails in different frequency ranges grouped by OSA risk based on STOP-BANG scores.

	OSA risk	Low/medium frequency		High frequency	
		0R(95%CI)	P value	0R(95%CI)	P value
Model 1	Low	ref		ref	
	Intermediate	2.04(1.25,3.33)	**<0.01** ^**^	1.78(1.28,2.48)	**<0.01** ^**^
	High	2.06(1.15,3.68)	**<0.05** ^*^	1.51(1.01,2.27)	**<0.05** ^*^
	Intermediate /high	2.05(1.32,3.17)	**<0.01** ^**^	1.68(1.25,2.24)	**<0.01** ^**^
Model 2	Low	ref		ref	
	Intermediate	1.12(0.64,1.95)	0.70	1.41(0.96,2.05)	0.08
	High	1.52(0.78,2.95)	0.22	1.52(0.96,2.42)	0.08
	Intermediate /high	1.23(0.74,2.06)	0.42	1.44(1.03,2.03)	**<0.05** ^*^
Model 3	Low	ref		ref	
	Intermediate	1.09(0.62,1.91)	0.76	1.38(0.94,2.01)	0.10
	High	1.45(0.74,2.85)	0.28	1.46(0.92,2.34)	0.11
	Intermediate /high	1.19(0.71,2.01)	0.51	1.41(1.00,1.98)	**<0.05** ^*^
Model 4	Low	ref		ref	
	Intermediate	1.06(0.59,1.90)	0.84	1.42(0.96,2.10)	0.08
	High	1.39(0.68,2.84)	0.37	1.46(0.89,2.42)	0.14
	Intermediate /high	1.14(0.66,1.98)	0.63	1.43(1.00,2.06)	**<0.05** ^*^

Range of low/medium frequency: 3 screening frequencies including 0.5, 1, and 2 kHz, Range of high frequency: screening frequency of 4 kHz.

*: P≤0.05 (Model 2 adjusted for age and BMI. Model 3 adjusted for age, BMI and smoke. Model 4 adjusted for age, BMI, hypertension, diabetes and dyslipidemia.)

## Discussion

In our study, it was revealed that the intermediate/high risk for OSA in middle-aged men assessed by STOP-BANG scores was associated with hearing impairment. Further analysis for subtypes of hearing impairment suggested a stronger association of intermediate/high risk for OSA with hearing impairment at high frequency of 4 kHz. After adjusting the potential cardiovascular risk factors, the association was still statistically significant.

The STOP-BANG questionnaire is reliable for screening patients with moderate to severe OSA. The risk of moderate to severe OSA is almost 0 in people with 0–2 points. The sensitivity of a score≥3 to detect OSA with apnea and hypopnea index >15 is 100%, but the specificity is only 43%. In order to improve the specificity, Chung et al [12] proposed a two-step method to include population with STOP scores ≥ 2 + BMI >35 kg/m^2^ or NC > 40cm besides STOP-BANG scores of 3–4 as a high-risk population. Then the specificity was increased to 79–85%. Those confirmed the reliability of STOP-BANG questionnaire to screen out the population with moderate to severe OSA. In our study, because individuals with BMI > 35kg /m^2^ were included in the population with STOP-BANG scores≥5, the STOP scores≥2 + NC > 40cm was used as the standard of two-step method. Individuals meeting the standard or with STOP-BANG scores ≥ 5 were classified as high risk for OSA.

Settings and environment for hearing screening test in our study are in line with the statement of ASHA’s adult hearing screening guidelines [14]. Hearing impairment is determined according to the 1997 grading standard for hearing loss by World Health Organization (WHO). This test is a desirable method for hearing screening in a large population, especially in the population accepting routine health check with a good health economic benefit for early detection of hearing loss. In our research population if individuals failed to respond to any pure tone at 25dB HL, they will be further referred to the full audiological assessment.

Prior studies on the relationship between OSA and hearing impairment revealed inconsistent results. Chopra et al [15] presented the association of OSA with hearing impairment in a Hispanic community population. After adjusting age and cardiovascular risk factors, they found subjects with severe OSA tended to have hearing impairment in high frequency range of 6 and 8 kHz, whereas those with moderate OSA tended to have hearing impairment at low frequency of 0.5 kHz. A stronger association of OSA with low frequency impairment was suggested. Kayabasi et al [16] found hearing impairment at high frequency of 4 and 8 kHz in patients with moderate OSA, while hearing impairment in all frequencies ranging from 0.25 to 8 kHz was found in severe OSA patients without adjusting cardiovascular risk factors. Matsumura and Li [17, 18] did not find any pure-tone audiometry abnormalities in patients with moderate and severe OSA, but the results of DPOAE showed severe OSA was associated with hearing abnormalities at frequencies of 1–8 kHz. It was attributed to the ability to detect early cochlear dysfunction by DPOAE. Martines et al [19] did not detect hearing impairment in simple snorers, whereas in patients with moderate/severe OSA a significant hearing impairment was shown in the high frequency range above 6 kHz, especially in the extended high frequency region of 10–16 kHz. The discrepant results of former studies may be due to the limited sample size, as well as different approaches to detecting OSA and hearing impairment. In our study, the intermediate/high risk for OSA was associated with hearing impairment mainly at 4 kHz. Since the predictive value of STOP-BANG scores mainly lies in screening for patients with moderate to severe OSA, the hearing impairment in the high-frequency range may be attributed to those with moderate to severe OSA included in the intermediate/high risk group. However, further studies should be conducted to confirm the diagnosis and degree of OSA by polysomnography.

It is speculated that OSA induces hearing loss through multiple mechanisms. Among the risk factors that lead to hearing loss, noise leads to high-frequency hearing loss, while hearing loss caused by cardiovascular risk factors such as smoking and diabetes involves both low and high frequency. Noise trauma leads to the loss of outer hair cells inside the Corti’s organ located in the basilar membrane of the cochlea, where high-frequency hearing is encoded. However, smoking and diabetes may lead to ischemia of the inner ear through the dysfunction of cochlear microcirculation and endothelial inflammation [20]. It may serve to aggravate hearing loss caused by noise. Whereas the hair cells which encode low-frequency hearing located at the apex of cochlear are more susceptible to ischemia. Severe OSA is considered to be a combined pathological damage including noise and hypoxia during sleep and commonly combined with cardiovascular risk factors [21]. Prior study reported poorer responses to steroid treatment for the improvement of hearing in patients with OSA and idiopathic sudden sensorineural hearing loss (ISSNHL) than patients without OSA. It was indicated that OSA-related hypoxia and snoring noise is hazardous to hearing and standard treatments with CPAP is suggested in OSA patients for both holistic and auditory health [22]. The rat experiment also proved the combination of noise and hypoxia will lead to 70–100% loss of outer hair cells in the high-frequency coding region, and even some loss of inner hair cells. The degree of hearing loss increases with the increasing degree of hypoxia and the extension of hypoxia time [23]. Besides these, ischemia-reperfusion injury and oxidative stress response may also contribute to the mechanisms elucidating the association between OSA and hearing impairment.

## Strength and limitations

Our study used reliable and easy to execute methods for assessing the risk for OSA and hearing impairment, which can be generalized for large-scale population study. We adjusted several confounding factors that may exist simultaneously in OSA and hearing loss. Our staff working on questionnaire collection and hearing screening test were blind of each other, and unaware of the research hypothesis, so the data were objective enough. Nevertheless, there were some limitations. 1. Future polysomnography should be conducted to explore the association between different degree of OSA and hearing impairment. 2. Pure-tone threshold testing can be used to identify the hearing impairment below 25 db HL.

## Conclusions

In conclusion, we found in Chinese midlife men, the intermediate/high risk for OSA was associated with hearing impairment, especially at the high frequency of 4 kHz. This finding reminded us of early identifying midlife men with OSA risk as a population at high risk for hearing impairment, hence offering proper intervention to prevent consequent deterioration of hearing loss.

## Supporting information

S1 Data (XLSX)
